# Rapid detection of three rabbit pathogens by use of the Luminex x-TAG assay

**DOI:** 10.1186/s12917-018-1438-8

**Published:** 2018-04-07

**Authors:** Miaoli Wu, Yujun Zhu, Feng Cong, Dan Rao, Wen Yuan, Jing Wang, Bihong Huang, Yuexiao Lian, Yu Zhang, Ren Huang, Pengju Guo

**Affiliations:** 1 Guangdong Provincial Key Laboratory of Laboratory Animals, Guangzhou, 510633 China; 2 Guangdong Laboratory Animal Monitoring Institute, Guangzhou, China; 3 Guangdong laboratory animals monitoring institute, Guangzhou, 510633 China; 4 Guangdong key laboratory of laboratory Animals, Guangzhou, China

**Keywords:** Rabbit hemorrhagic disease virus, Rabbit rotavirus, Sendai virus, Multiple PCR, Luminex x-TAG

## Abstract

**Background:**

Domestic rabbits especially New Zealand white rabbits play an important role in biological research. The disease surveillance and quality control are essential to guarantee the results of animal experiments performed on rabbits. Rabbit hemorrhagic disease virus, rabbit rotavirus and Sendai virus are the important pathogens that needed to be eliminated. Rapid and sensitive method focus on these three viruses should be established for routine monitoring. The Luminex x-TAG assay based on multiplex PCR and fluorescent microsphere is a fast developing technology applied in high throughput detection. Specific primers modified with oligonucleotide sequence/biotin were used to amplify target fragments. The conjugation between oligonucleotide sequence of the PCR products and the MagPlex-TAG microspheres was specific without any cross-reaction, and the hybridization products could be analyzed using the Luminex 200 analyzer instrument. Recombinant plasmids were constructed to estimate the detection limit of the viruses. Furthermore, 40 clinical samples were used to evaluate the efficiency of this multiplex PCR based Luminex x-TAG assay.

**Results:**

According to the results, this new method showed high specificity and good stability. Assessed by the recombinant plasmids, the detection limit of three viruses was 100copies/μl. Among 40 clinical specimens, 18 specimens were found positive, which was completely concordant with the conventional PCR method.

**Conclusions:**

The new developed Luminex x-TAG assay is an accurate, high throughput method for rapid detection of three important viruses of rabbits.

## Background

Domestic rabbit (*Oryctolagus cuniculus*), especially New Zealand white rabbit, has attracted more and more attention in biomedical, immunological and pharmaceutical research, because of its intermediate size and phylogenetic proximity to primates [1]. It played an important role in production of antibodies, eye research as well as cardiovascular disease [2, 3]. Rabbit is one of the most commonly used experimental animals and must be free of some important pathogens.

The first outbreak of rabbit hemorrhagic disease (RHD) caused by the rabbit hemorrhagic disease virus (RHDV) occurred in 1984 in Jiangsu Province, China and spread all around the world rapidly [4]. It’s an acute and mostly fatal contagion in both domestic and wild rabbits, characterized by acute necrotizing hepatitis and hemorrhage [5]. Actually there are three different clinical features, the pre-acute, acute and sub-acute forms [6]. Among which the sub-acute form causes no clinical symptoms and rabbits will recover within 2~ 3 days [7]. Rabbit rotavirus (RRV) infection was the major cause of mild to severe diarrhea in rabbits [8]. The rotavirus isolated from infected rabbits belongs to Group A rotaviruses (RVAs), which also infect humans and other animals. It’s a highly contagious mild virus and disseminated by fecal-oral route [9–11]. Although the infection rate of RRV is high, most infections are subclinical. However, co-infection with other bacteria or viruses may cause severe enteritis and the excretion by the infected rabbits will become the contaminate source and cause new infection. Sendai virus (SV), also known as a murine parainfluenza virus type 1, belongs to *Respirovirus*, *Paramyxoviridae* family. It causes transmitted respiratory tract infections in a variety of animals [12]. Unlike rodents, rabbits are not sensitive to SV, and the infection will only cause fever but not respiratory tract contagious in rabbits.

Despite the asymptomatic infection and low mortality of rotavirus and Sendai virus infection, the existence of these two viruses will affect the quality of experimental animals and severely interfere with the results of animal experiments on them [13]. To improve the quality of rabbits and ensure the accuracy of animal experiments, RHDV, SV and RRV are the required inspection items ruled by the national quality standard of China.

The traditional methods for pathogen identification include etiology diagnosis, serological diagnosis as well as molecular diagnosis [14, 15]. According to the laboratory animal microbiological quality control standards of China, the recommended test methods for these viruses mainly are the etiology and serological diagnosis. Both of them are time-consuming and laborious, compared with molecular diagnostic techniques. Polymerase chain reaction (PCR) with high sensitivity and specificity is widely used in pathogeny identification [16, 17]. Reverse transcription-PCR (RT-PCR) and quantitative reverse transcription-PCR (RT-qPCR) assays had been developed for monitoring of rabbit hemorrhagic disease virus, Sendai virus as well as rabbit rotavirus [18–20]. However, the restricted throughput limited the application of PCR, even the multiplex real-time quantitative PCR could not detect more than five pathogens in one reaction. The development of rapid and sensitive multiplex diagnostic method was extremely important for rabbit health monitoring. Compared with conventional PCR methods, the Luminex technology was a high-throughput, rapid, sensitive and labor-saving multiplex assay [21]. Conjugation of microbeads with different fluorescent dyes could differentiate as much as 100 targets in a single reaction. This technology offered a variety of applications in pathologic diagnosis [22–24].

In this study, we developed a multiplex PCR-based MagPlex-TAG assay for simultaneous detection of rabbit hemorrhagic disease virus, rabbit rotavirus and Sendai virus.

## Methods

### Virus and vaccine

The combined rabbit viral hemorrhagic disease and *Pasteurrella* multocida vaccine and rabies vaccine were purchased from the animal epidemic prevention and control center in Tianhe district (Guangzhou, China). The nucleic acid of classical RHDV strain was kindly offered by Shanghai Veterinary Research Institute. The Sendai virus, *Salmonella typhimurium*, *Helicobacter bilis* (H.b), *Helicobacter rodent* (H.r), *Escherichia coli* (E.coli) and the nucleic acid of rabbit rotavirus, rabbit adenovirus as well as rabbit coronavirus were preserved by our laboratory.

### Sample collection and DNA/RNA extraction

All the clinical samples, including 24 fecal samples, 10 tissue samples and 6 nose swabs, were provided by a rabbit farm from Qingdao (Shandong Province, China) and preserved at − 80 °C. The nucleic acid extraction was performed by the automatic nucleic acid extraction instrument (Tiangen Biotech, Beijing, China) according to the manufacture’s instruction. All the nucleic acid were stored at − 80 °C before used.

### Primers design

Specific primers of the three viruses were designed based on the conserved VP60 gene, N gene and VP4 gene of rabbit hemorrhagic disease virus, Sendai virus and rabbit rotavirus respectively (Table 1). Each forward primer was modified with a unique oligonucleotide “TAG” sequence at the 5′ terminus, which was used to couple with the magnetic fluorescent microsphere. All the reverse primers were biotinylated at the 5′ terminus for fluorescent detection with streptavidin-R-phycoerythrin (SAPE). The forward primers and oligonucleotides were best match by the Tag-It Oligo Design Software v.3.00 (7B052). All the primers were synthesized and purified by high-performance liquid chromatography (HPLC) (Sangong, Guangzhou, China).

**Table 1:** Sequences of primers used in the Luminex x-TAG assay

Primer	Sequence	Target gene	Accession number	Genome location	Product size (bp)
RHDV-F	CTCTCCACAAAATAACCCATTCACA	VP60	KF494951.1	285~ 309	161
RHDV-R	CCAACCCTGGTCCAATCTCG			445–426	
RRV-F	ATGGTTCGCTTGTGTCTTAGTTG	VP4	U62152.1	303~ 325	251
RRV-R	ATGCGTTGGGTGTAGTTCCTGTA			553~ 531	
SV-F	TGACAACAAACGGAGTAAACGC	N	AB753448.1	269~ 289	148
SV-R	ACCATAGGTCCAAACAGCCATTC			416~ 394	

### Multiplex PCR amplification

Three sets of primers were confirmed by the simplex PCR reactions before the multiplex PCR (mPCR) amplification. The mPCR was performed using the one-step RT-PCR Kit (Qiagen, Valencia, CA; Cat.no.210212) with a total volume of 50 μl containing 100 ng of template DNA/RNA. The mPCR amplification conditions were as follows: 50 °C, 30 min for reverse transcription, followed by activation at 95 °C for 15 min. Then, DNA amplification was carried out by 35 cycles of 94 °C for 30 s, 60 °C for 30 s and 72 °C for 30 s, followed by final extension at 72 °C for 10 min. All the samples were tested in triplicate and the assays were run with negative control.

### Luminex assay

The PCR products were conjugated with MagPlex-TAG microspheres, which were pre-coupled with “anti-TAG” sequence. The working mixture containing 2500 of each target microspheres was diluted with 1× Tm Hybridization Buffer (0.2 M NaCl, 0.1 M Tris, 0.08% Triton X-100, Ph 8.0, filter sterilized). For each reaction, 5 μl of amplified product or distilled water, 75 μl of SAPE solution and 20 μl of the working MagPlex-TAG microsphere mixture were well mixed together before incubated in a thermocycler for 30 min at 45 °C. The Luminex 200 analyzer was applied to analyze the products after the hybridization reaction.

### Data analysis

The data analyzed by the Luminex xPONENT software were reported as median fluorescence intensity (MFI). For background calculation, negative controls contained all the hybridization components except target DNA, were set in each experiment. The cutoff value of the three target pathogens were obtained from all the negative PCR controls. Specifically, the cutoff value was defined, for each bead set as the mean of the MFI values of negative controls + 3 SD (Standard deviation).

### The evaluation of specificity

To evaluate the specificity of the x-TAG assay, the *Pasteurella* (Pas.), *Salmonella typhimurium* (S.ty), *Helicobacter bilis* (H.b), *Helicobacter rodent* (H.r), *Escherichia coli* (E.coli), rabbit coronavirus (RCoV), rabbit adenovirus (RAV) and rabies virus (RV) nucleic acids were tested with previously indicated primers. Positive and negative controls were simultaneously tested. All the products were further analyzed on the Luminex 200 analyzer after hybridization.

### The evaluation of sensitivity

The PCR products of RHDV (161 bp), RRV (251 bp) and SV (148 bp) were cloned into the pGEM T easy vector (Promega, Madison, USA). The plasmids were in vitro transcribed (IVT) by RiboMax™ Large Scale RNA production system T7 (Promega, Madison,USA) according to the manufacturer’s instructions. 40 units RNase-free DNase™ (Promega, Madison, USA) enzyme was used to remove plasmid DNA. Trizol LS reagent (Invitrogen, Carlsbad, CA) was used for RNA isolation according to the manufacturer’s instructions. The RNA concentrations were estimated by spectrophotometry. Serial ten-fold dilutions of RNA standard were performed as templates to determine the sensitivity of the x-TAG assay, and the results were confirmed in triplicate.

### Clinical samples detection

A total number of 40 clinical samples including nose swabs, faeces and tissues were tested by both Luminex x-TAG assay and conventional PCR assay.

## Results

### Specificity analysis of the Luminex x-TAG assay

**Fig. 1:**
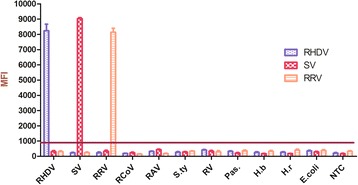
Result of the specificity analysis. Each bar represents the average median fluorescence intensity (MFI) of duplicate samples with standard deviation. The cut-off value was about 1000, calculated by the formula: cut-off value = mean MFI values of negative controls + 3 SD (Standard Deviation). Distilled water was used as the negative control (NTC). RHDV: rabbit hemorrhagic disease virus; SV: Sendai virus; RRV: rabbit rotavirus; RCov: rabbit coronavirus; RAV: rabbit adenovirus; S.ty: *Salmonella typhimurium*; *Pas*.: *Pasteurella*; H.b: *Helicobacter bilis*; H.r: *Helicobacter rodent*; E.coli: *Escherichia coli*; RV: rabies virus

The specificity of the three primer pairs was confirmed using the unrelated nucleic acid as PCR templates. There was no cross-amplification during the test and the fluorescence signals were observed only in the corresponding positive controls (Fig.1).

### Sensitivity of the Luminex x-TAG assay

**Fig. 2:**
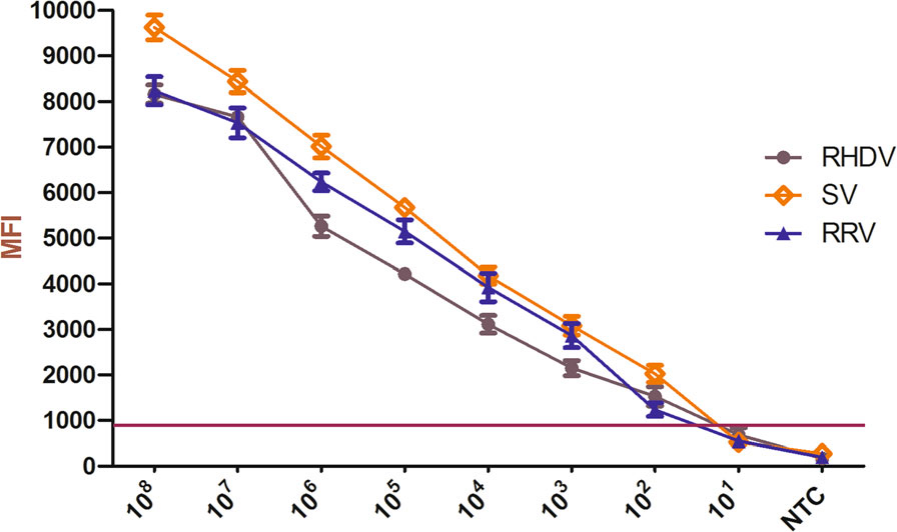
Result of the sensitivity analysis. The concentration of standard RNA ranged from 10^8^copies/μl to 10^1^copies/μl. NTC represented the negative control. All the samples were tested in triplicate. The cut-off value was 1000 and the detection limit of three viruses was 10^2^copies/μl

The sensitivity of the Luminex x-TAG assay was examined by testing serial ten-fold dilutions of RNA standard, and distilled water was used as negative control. The results showed that the detection limits of the three viruses were 10^2^copies/μl (Fig. 2). The MFI value and the corresponding concentration were detailed in Table 2.

**Table 2:** The MFI values of different standard RNA concentrations

Sample concentration	RHDV		SV		RRV	
	Mean MFI	SD	Mean MFI	SD	Mean MFI	SD
10^8^ copies/μl	8159	196	9621	215	8324	245
10^7^ copies/μl	7639	134	8421	195	7502	227
10^6^ copies/μl	5256	221	7026	143	6242	194
10^5^ copies/μl	4211	115	5673	136	5103	187
10^4^ copies/μl	3106	194	4168	185	3965	278
10^3^ copies/μl	2154	164	3075	204	2837	243
10^2^ copies/μl	1518	103	1946	184	1264	149
10^1^ copies/μl	572	117	543	125	496	138
Background	174	29	267	35	185	26
Cut-off value	1000		1000		1000	

### Reproducibility and stability analysis

To further assess the Luminex x-TAG assay, three parallel reactions were carried out with standard RNA at concentrations of 1 × 10^5^ and 1 × 10^8^ copies/μl. The coefficient of variation of the intra-assay and inter-assay were displayed in Table 3.

**Table 3:** The reproducibility and stability analysis of the Luminex x-TAG assay

Virus	concentration (copies/μl)	Intra-assay/MFI			CV(%)	Inter-assay/MFI			CV(%)
		1	2	3		1	2	3	
RHDV	1 × 10^8^	8086	8381	8012	2.4	8321	8075	8112	1.62
	1 × 10^5^	4081	4284	4271	2.73	4405	4312	4165	2.81
SV	1 × 10^8^	9414	9629	9821	2.23	9632	9563	9412	2.21
	1 × 10^5^	5723	5775	5518	2.4	5583	5695	5354	3.13
RRV	1 × 10^8^	8441	8052	8478	2.94	8446	8249	8056	2.36
	1 × 10^5^	5218	5194	4907	3.67	5048	5421	5339	3.71

### Application to clinical samples

The Luminex x-TAG assay was applied for clinical sample detection, and confirmed by both the conventional PCR and sequencing analysis. The assay detected genetic material of RHDV (*n* = 3), SV (*n* = 15) and RRV (*n* = 10) in the clinical samples. Among which, the co-infection of RHDV +SV (*n* = 2), RHDV+RRV (*n* = 3), SV + RRV (*n* = 7) was observed. Only 2 samples were detected with triple infection (Table 4). All the samples were detected by conventional PCR method and the results were in accord with the Luminex x-TAG assay.

**Table 4:** Screening results for 40 clinical samples for Luminex x-TAG assay

Sample	RHDV	SV	RRV	Sample	RHDV	SV	RRV
F1	–	+	+	F21	–	+	–
F2	–	–	–	F22	–	–	–
F3	–	–	–	F23	–	–	–
F4	–	+	–	F24	–	+ +	+
F5	–	+	–	T1	–	+	–
F6	–	–	–	T2	+	–	+
F7	–	–	–	T3	–	–	+ +
F8	–	–	–	T4	+ +	+	+
F9	–	+	–	T5	+	+ +	+ +
F10	–	–	–	T6	–	+ +	+
F11	–	–	–	T7	–	+	+
F12	–	–	–	T8	–	–	–
F13	–	–	–	T9	–	–	–
F14	–	–	–	T10	–	+	–
F15	–	–	+ +	N1	–	–	–
F16	–	–	–	N2	–	–	–
F17	–	+	–	N3	–	–	–
F18	–	–	–	N4	–	–	–
F19	–	+ +	+	N5	–	+ + +	–
F20	–	–	–	N6	–	–	–

*F* faecal samples, *T* tissue specimens, *N* nose swab

+ + +: strong positive (MFI > 5* cut-off)

+ +: positive (3* cut-off < MFI < 5* cut-off)

+: weak positive (cut-off < MFI < 3* cut-off)

-: negative (MFI < cut-off)

## Discussion

Although conventional PCR had been used for RHDV, SV and RRV identification, there is no multiplex-assay for simultaneous discrimination of these viruses. Multiplex-PCR assays allow for detection of different targets in one reaction. The interpretation of the mPCR results is based on the sizes of amplicons. It limits the multiplicity in the single reaction and fails to meet the requirements of hig- throughput detection.The Luminex x-TAG assay based on PCR products coupled with fluorescent encoding microsphere is a high-throughput, accuracy technique, which is widely used in pathogenic diagnosis [25]. However, there is a lack of studies that apply and investigate x-TAG assay in the veterinary field.

In this study, we described the development and validation of the Luminex x-TAG assay for monitoring of RHDV, SV, and RRV. The new method showed good specificity, no cross-reaction with other tested pathogens. The detect limit of the three viruses was 10^2^ copies/μl. 40 specimens were tested using both Luminex x-TAG assay and conventional RT-PCR, and the results of different methods are consistent with each other. These findings suggest that the developed x-TAG assay based on multiplex PCR for screening three pathogens is applicable.

To further optimize the assay, we performed it on different hybridization conditions.The results indicated that 45 °C was the optimal hybridization temperature and the addition of 1% BSA in the 1× Tm Hybridization Buffer could remarkably reduce the background MFI values.

A large scale of negative samples mainly SPF samples should be tested to determine the cut-off value of each viruses. To guarantee the accuracy of the test and avoid false positive/negative results, MFI values of specimen that is close to the threshold should be double-checked by monoplex PCR or sequencing.

## Conclusion

The multiplex assay is an efficient alternative to monoplex RT-PCR and greatly reduces the number of reactions required. In this report, the establishment of this effective system will allow precise detection and identification of RHDV,SV and RRV. This approach might be the promising rabbit quality control methods. Besides, more target pathogens could be integrated into the established assay for better utilization.

## Abbreviations

E.coli: *Escherichia coli*
H.b: *Helicobacter bilis*
H.r: *Helicobacter rodent*
NTC: Non template control
Pas.: *Pasteurrella multocida*
RAV: Rabbit adenovirus
RCov: Rabbit Coronavirus
RHD: Rabbit hemorrhagic disease
RHDV: Rabbit hemorrhagic disease virus
RRV: Rabbit rotavirus
RV: Rabies virus
RVAs: Group A rotaviruses
S.ty: *Salmonella typhimurium*
SV: Sendai virus
